# Community structure and functional group of root‐associated Fungi of *Pinus sylvestris* var. *mongolica* across stand ages in the Mu Us Desert

**DOI:** 10.1002/ece3.6119

**Published:** 2020-02-19

**Authors:** Pei‐shan Zhao, Mi‐shan Guo, Guang‐lei Gao, Ying Zhang, Guo‐dong Ding, Yue Ren, Mobeen Akhtar

**Affiliations:** ^1^ Yanchi Research Station School of Soil and Water Conservation Beijing Forestry University Beijing China; ^2^ Key Laboratory of State Forestry and Grassland Administration on Soil and Water Conservation Beijing China; ^3^ Engineering Research Center of Forestry Ecological Engineering Ministry of Education Beijing Forestry University Beijing China

**Keywords:** ectomycorrhizal fungi, pathogenic fungi, *Pinus sylvestris* var. *mongolica*, saprotrophic fungi, soil property, stand age, the Mu Us Desert

## Abstract

Root‐associated fungi (RAF) are an important factor affecting the host's growth, and their contribution to *Pinus sylvestris* var. *mongolica* plantation decline is substantial. Therefore, we selected three age groups of *P. sylvestris* plantations (26, 33, and 43 years), in the Mu Us Desert, to characterize the community structure and functional groups of RAF, identified by Illumina high‐throughput sequencing and FUNGuild platform, respectively. The effects of soil properties and enzyme activities on fungal diversity and functional groups were also examined. The results indicated that (a) 805 operational taxonomic units of RAF associated with *P. sylvestris* belonged to six phyla and 163 genera. Diversity and richness were not significantly different in the three age groups, but community composition showed significant differences. Ascomycota and Basidiomycota dominated the fungal community, while *Rhizopogon* dominated in each plot. (b) The proportion of pathotrophs decreased with increasing age, while that of symbiotrophs increased sharply, which were mainly represented by ectomycorrhizal fungi. (c) Stand age and soil enzyme activity had a greater influence on fungal community composition than did soil properties, whereas environmental variables were not significantly correlated with fungal diversity and richness. Dynamics of fungal community composition and functional groups with the aging plantations reflected the growth state of *P. sylvestris* and were related to plantation degradation.

## Graphical abstract

In this article, the community structure and functional groups of root‐associated fungi of *Pinus sylvestris* var. *mongolica* plantations with different stand age (26, 33 and 43 years) in the Mu Us Desert were identified by Illumina high‐throughput sequencing and FUNGuild platform. Dynamics of fungal community composition and functional groups with the ageing plantations reflected the growth state of *P. sylvestris* and were related to plantation degradation.

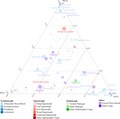

## 1 INTRODUCTION

*Pinus sylvestris* var. *mongolica* plantations are diminishing in the desertified Northern China.

As an important evergreen species with strong environmental resistance, millions of *P. sylvestris* have been planted in the desertified Northern China, to protect meadows, farmlands, oases, etc. Presently, *P. sylvestris* plantations occupy at least 3.0 × 10^5^ ha in the desertified land (Song et al., 2018). *P. sylvestris* alleviates the effects of desertification and sandstorms, mainly by reducing the wind speed and enhancing sand fixation. Both the ecosystems and citizens benefit from these green guards. However, these *P. sylvestris* plantations have been experiencing leaf etiolation, disease outbreaks, growth declines, and regeneration barriers since the 1990s (Song, Zhu, Yan, Li, & Yu, 2015). The life span of *P. sylvestris* plantations is about 60 years, which is much shorter than that of natural forests, and the quality of these plantations has significantly declined in the past few years (Zhu, Fan, Zeng, Jiang, & Matsuzaki, 2003). The degradation of these plantations is continuing and is spreading to other maturing *P. sylvestris* plantations in the desertified lands of Northern China. Therefore, there is an urgent need to identify the causes underlying this deterioration.

Due to the aridity and water shortage in the desertified regions, water stress severely restricts plant growth (Liu, Bao, Song, Cai, & Sun, 2009). Hence, it has been suggested that soil moisture is the critical factor determining *P. sylvestris* plantation degradation, because variations in precipitation and available groundwater result in an unsustainable water balance (Song, Zhu, Li, & Zhang, 2016; Zheng, Zhu, Yan, & Song, 2012). However, in practice, although the increasing rainfall and careful irrigation have greatly improved the *P. sylvestris* plantation quality, forest degradation still restricts the development and benefits of *P. sylvestris* plantations, especially their regeneration. Previous studies have proven that rainfall exhibits significant secular trends in the semi‐arid areas of Northern China (Gong, Shi, & Wang, 2004; Wei & Wang, 2013); however, the plantations continue to recede, indicating that some unheeded and invisible factors are driving this process.

Root‐associated fungi (RAF), which form a large invisible world beneath the soil, connect the aboveground biomass with the underground ecosystem. They play important roles in ecosystem functions, by participating in a series of crucial ecological processes in terrestrial ecosystems (Fisher et al., 2012; Wehner et al., 2014). In early studies, scholars mainly focused on RAF diversity and community composition in specific habitats or host plants (Barnes, Maldonado, Froslev, Antonelli, & Ronsted, 2016a; David, Seabloom, & May, 2016). The identification of fungal functional groups was then explored, based on RAF community taxonomy, to accurately reflect the growth, development, and fitness of host plants (Nguyen et al., 2016). The connection between RAF of different functional groups and hosts can be both beneficial and detrimental. Pathogenic fungi can cause diseases in host plants, while symbiotic fungi promote the absorption of water and mineral nutrients by the host plants, and improve resistance and adaptability (Kolaříková et al., 2017). Compared to saprotrophic fungi, the productivity and diversity of plant communities respond more strongly to the richness of ectomycorrhizal (ECM) and plant pathogenic fungi (Peay, Baraloto, & Fine, 2013). Therefore, functional groups provide a better and direct understanding of the correlations between visible vegetation and invisible soil microbes, suggesting that these fungi may be potential decisive factors for *P. sylvestris* plantation degradation.

Root‐associated fungi community structure and functional groups are influenced by host characteristics and the abiotic environment. In secondary forests with various plant species, host specificity or preference is a key driver (Horn, Hempel, Verbruggen, Rillig, & Caruso, 2017), but in case of a single target plant species, stand age has been shown to be an important determinant of the fungal community composition (Gao et al., 2014; Spake et al., 2016). Moreover, soil properties are also regarded as crucial environmental variables. The communities and trophic modes of RAF may fluctuate with the pH (Li et al., 2018), water content (Barnes, Gast, Burns, McNamara, & Bending, 2016b), elemental composition (Liu et al., 2014), and soil enzyme activities (Kyaschenko, Clemmensen, Hagenbo, Karltun, & Lindahl, 2017). However, it is challenging to determine the causalities between soil factors and fungi assembly. A comprehensive and reasonable interpretation and understanding of the RAF community and functional groups need to be fully combined with a variety of driving factors.

The Mu Us Desert, located in Northern China, is one of the twelve largest deserts in China, occupying a total area of 3.2 km^2^ across the Shaanxi Province and the Inner Mongolia and Ningxia Hui Autonomous Regions. As an optimal species for conifer afforestation, *P. sylvestris* was successfully introduced into Yulin City, Shaanxi Province, from the Hulunbuir Desert, in 1964. Since then, *P. sylvestris* plantations have continued to increase, albeit accompanied by forest degradation. In this paper, we hypothesized that *P. sylvestris* plantation degradation is a consequence of variations in RAF and their functions, resulting from stand aging. The fungal community structure was examined across a chronosequence of stand age, for 26 (half‐mature forest), 32 (nearly mature forest), and 43 (mature forest) years, and functional groups were analyzed using the FUNGuild platform. The objectives of this study were as follows: (a) to explore the variations in community structure and functional groups of RAF of *P. sylvestris*, (b) to identify how the soil factors and stand age affect these variations. This information will improve our knowledge of *P. sylvestris* plantation degradation, and provide a firm basis for drafting and implementing policies for efficiently restoring degraded plantations.

## 2 MATERIALS AND METHODS

### 2.1 Study site

The study site was located in the Hongshixia Sandy Botanical Garden, on the southern edge of the Mu Us Desert (38°26′N, 109°12′E; 1,080 m elevation), Shaanxi Province. The site had a warm temperate continental monsoon climate, characterized by four distinct seasons, adequate light and heat, and great temperature variations. The annual average temperature was 9.1°C, and the annual average precipitation and evaporation were 385.5 and 2,502 mm, respectively. The soil was classified as azonal eolian sandy soil, with loose topsoil structure and low water retention (Gao et al., 2014). The predominant vegetation included *P. sylvestris*, *Artemisia sacrorum*, *Tribulus terrestris*, *Bidens pilosa*, and *Agropyron cristatum*.

We selected three 50 × 50 m plots in *P. sylvestris* plantations under different stand ages: (a) half‐mature forest (MUh), (b) nearly mature forest (MUn), and (c) mature forest (MUm). All plots were established without tending measures and visible disturbance by anthropogenic activities. Information regarding the plots, including a substantial range of basic plot investigation, soil enzyme activities, and soil properties, was assessed (Table 1; Table S1).

**Table 1:** Basic characteristics of different age groups

Plot	Stand age (a)	Average height (m)	Average DBH (cm)	Stand density (N/ha)	Canopy density
MUh	26	12.48 ± 3.69	11.76 ± 3.72	2,500	0.79
MUn	32	13.96 ± 2.38	13.58 ± 2.44	2,500	0.86
MUm	43	14.14 ± 1.84	19.95 ± 3.03	1,650	0.73

Note

Values are mean ± *SE*.

Abbreviation: DBH, diameter at breast height.

### 2.2 Sample collection

Samples were collected in August 2017, which is the peak of the growth season. Within each age group, five standard trees, at least 10 m apart, were sampled. At each sampling position, litter, herbs, and the undergrowth humus layer were removed, and terminal root samples were dug out, careful not to include any miscellaneous roots. Then, the three repeated samples in the same tree were mixed to create one composite sample. In addition, soil around the root system (about 0–20 cm deep), corresponding to each root sample, was collected independently to evaluate soil properties. A total of 15 fine roots samples (three plots × 5 standard trees) were collected and prepared for subsequent assays. All samples were placed in sealed bags and preserved at −4°C.

### 2.3 Soil analysis

The soil water content (SWC) used to be determined gravimetrically after drying samples in an oven at 105°C for 12 hr. Soil pH was measured using a PHS‐3E pH meter (INESA, Shanghai, China). Total nitrogen (TN) and total phosphorus (TP) content were measured with the indophenol blue spectrophotometric method and Mo‐Sh anti‐colorimetric analysis method, respectively, with SmartChem Discrete Auto Analyzer (AMS). The total soil organic carbon (SOC) was determined using the dichromate oxidation method. Soil ammonium (NH_4_^+^‐N) and nitrate (NO_3_^−^‐N) were extracted with 2 M KCl solution and quantified colorimetrically from the supernatant using TU‐1810 UV‐Vis spectrophotometer (Pgeneral). Invertase, urease, and acid phosphatases activity levels were measured by 3,5‐dinitrosalicylic acid colorimetry, sodium phenol‐sodium hypochlorite colorimetry, and phenyl phosphate disodium salt colorimetry, respectively.

### 2.4 Sequencing data and fungal functional groups

DNA was extracted using the PowerSoil^®^ DNA Kit (MO BIO), according to the manufacturer's protocol. Polymerase chain reaction (PCR) amplification of RAF rDNA internal transcribed spacer (ITS) was performed, using the primers ITS1F (5′‐CTTGGTCATTTAGACGAAGTAA‐3′) and ITS2 (5′‐GCTGCGTTCTTCATCGATGC‐3′). The PCR amplification products were then detected and quantified using AXYGEN Gel Extraction Kit (Qiagen) and QPCR, respectively. An equimolar mix of all three amplicon libraries was used for sequencing, at the Allwegene Company. Each sequence was separately used to perform individual nucleotide–nucleotide searches, using the NCBI BLAST algorithm (http://blast.ncbi.nlm.nih.gov/Blast.cgi), against the fungal taxa. Sequences with 97% similarity were classified as an operational taxonomic unit (OTU).

The functional groups of RAF in our study were assessed using the FUNGuild platform (http://www.stbates.org/guilds/app.php) (Nguyen et al., 2016), and only results with guild assignments that were “highly probable” or “probable” were accepted (Table 2). It was identified that fungi with combined trophic mode and combined guild were incorporated into “other fungi” and “other pathotrophic/saprotrophic fungi,” respectively.

**Table 2:** Classification of fungal trophic modes and guilds

Trophic mode	Guild
Pathotroph	Animal pathogens
	Plant pathogens
	Other pathotrophic fungi
Saprotroph	Dung saprotrophs
	Plant saprotrophs
	Soil saprotrophs
	Wood saprotrophs
	Undefined saprotrophs
	Other saprotrophic fungi
Symbiotroph	Arbuscular mycorrhizal fungi
	Ectomycorrhizal fungi
	Endophytes
	Lichenized fungi
Other fungi	–

### 2.5 Statistical analyses

Shannon, Pielou, and Simpson indices were calculated, based on the relative abundance of OTUs, using the Vegan package in R‐3.6.0. One‐way analysis of variance (ANOVA) with least‐significant difference (LSD) was used to compare the diversity indices and soil properties among the three age groups. All statistical analyses were performed in SPSS 20.0, and *p* < .05 was considered statistically significant.

For the convenience of description, the RAF genera are divided into dominant genus (>10.00%), common genus (10.00% ~ 1.00%), and rare genus (<1.00%) according to relative abundance. Groups of RAF (relative abundance > 1.00%) and intergroup similarity are demonstrated by a cluster heatmap using the Ward method and Euclidean distance, and cluster method, respectively. Fungal community memberships and their distribution patterns in different age groups were shown in a ternary plot. For this, relative abundances of the genera and OTUs were log‐transformed prior to analyses, to improve variance homogeneity. The cluster heatmap and ternary plot were both created in OriginPro 2018.

Mantel test, Spearman's rank correlation analysis, and redundancy analysis (RDA) were employed to explain the relative roles of soil variables in fungal community composition. Prior to the Mantel test, a Bray–Curtis dissimilarity index was calculated to examine the dissimilarity of the OTU communities between sites, and dissimilarity matrices of the environmental data were calculated based on Euclidean distance. Pearson's product‐moment correlation was applied, and the significance level was examined using a permutation method with 999 repetitions. Spearman's rank correlation analysis was used to determine the correlations between diversity, richness, soil properties, and soil enzyme activities. Subsequently, RDA was used to determine the correlation between soil properties, soil enzyme activities, and functional groups, and the significance of each environmental variable was analyzed by Monte Carlo permutation test (999 permutations). The indicator RAF genera were identified to further assess the distribution of RAF in different age groups. Mantel test and the filter of indicator genus were implemented using the “vegan” and “labdsv,” respectively, in R‐3.6.0. Spearman's rank correlation analysis and RDA were performed in SPSS 20.0 and Canoco 4.5, respectively.

## 3 RESULTS

### 3.1 Fungal diversity and community structure

We obtained a total of 361,548 high‐quality sequences after processing and comparing against our criteria, which were clustered into 805 OTUs. The alpha diversity indices were not significantly different between the stand ages (*p* < .05) (Table 3). Shannon, Simpson, and Pielou indices exhibited maximum values in the half‐mature forest, which were 3.10, 0.90 and 0.55, respectively.

**Table 3:** Diversity and richness of RAF in different age groups

	Shannon index	Simpson index	Pielou index	Richness index
MUh	2.97 ± 0.45 a	0.86 ± 0.07 a	0.52 ± 0.08 a	315.60 ± 23.13 a
MUn	3.10 ± 0.22 a	0.90 ± 0.02 a	0.55 ± 0.03 a	293.60 ± 60.98 a
MUm	3.03 ± 0.34 a	0.88 ± 0.06 a	0.54 ± 0.06 a	280.00 ± 31.24 a

Note

Values are mean ± *SE*. Same minuscule alphabet in column indicates nonsignificant divergence.

**Figure 1:**
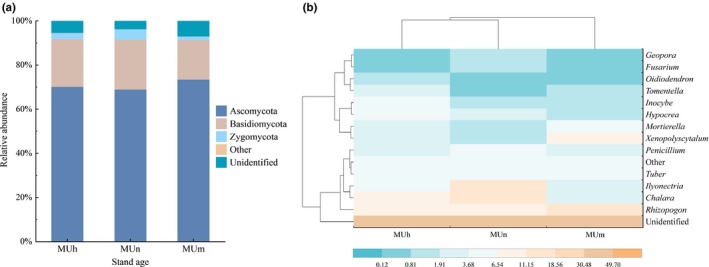
Taxonomic distribution and relative abundance of RAF in different age groups. (a) phylum‐level (>1.00%) and (b) genus‐level (>1.00%). The relative abundance of phylum/genus < 1.00% was counted as “other.”

Operational taxonomic units were assigned to six phyla, 17 classes, 54 orders, 87 families, and 163 genera. Across all samples, not only the relative abundance of Ascomycota (69.00%–73.50%) was higher than Basidiomycota (17.92%–22.60%) (Figure 1(a)), but Ascomycota also contain more genera. Among all the dominant and common genera, *Chalara*, *Ilyonectria*, *Tuber*, *Xenopolyscytalum*, *Penicillium*, *Hypocrea*, *Oidiodendron*, *Fusarium*, and *Geopora* belong to Ascomycota, while only three genera were assigned to Basidiomycota (*Rhizopogon*, *Inocybe,* and *Tomentella)*. Other phyla included Rozellomycota, Chytridiomycota, and Glomeromycota. There was no significant difference of relative abundance in phylum‐level between age groups (*p* > .05).

In genus‐level, the dominant fungi were different in each plot (Figure 1(b)). There were no dominant genera in the half‐mature forest, and the common genera, such as *Rhizopogon* (7.24%), *Chalara* (6.84%), *Inocybe* (5.58%), and *Hypocrea* (5.24%), were distributed evenly. Conversely, in nearly mature forests, *Chalara* (12.28%), *Ilyonectria* (11.27%), and *Rhizopogon* (10.96%) occupied a dominant position, followed by *Penicillium* (4.22%) and *Tuber* (4.05%). In mature forests, the genus with the highest relative abundance was *Rhizopogon* (17.05%), followed by *Xenopolyscytalum* (7.44%), *Mortierella* (4.67%), and *Tuber* (4.42%). The RAF community structure, based on genera, was more similar between the half‐mature and nearly mature forests.

### 3.2 Functional groups

**Figure 2:**
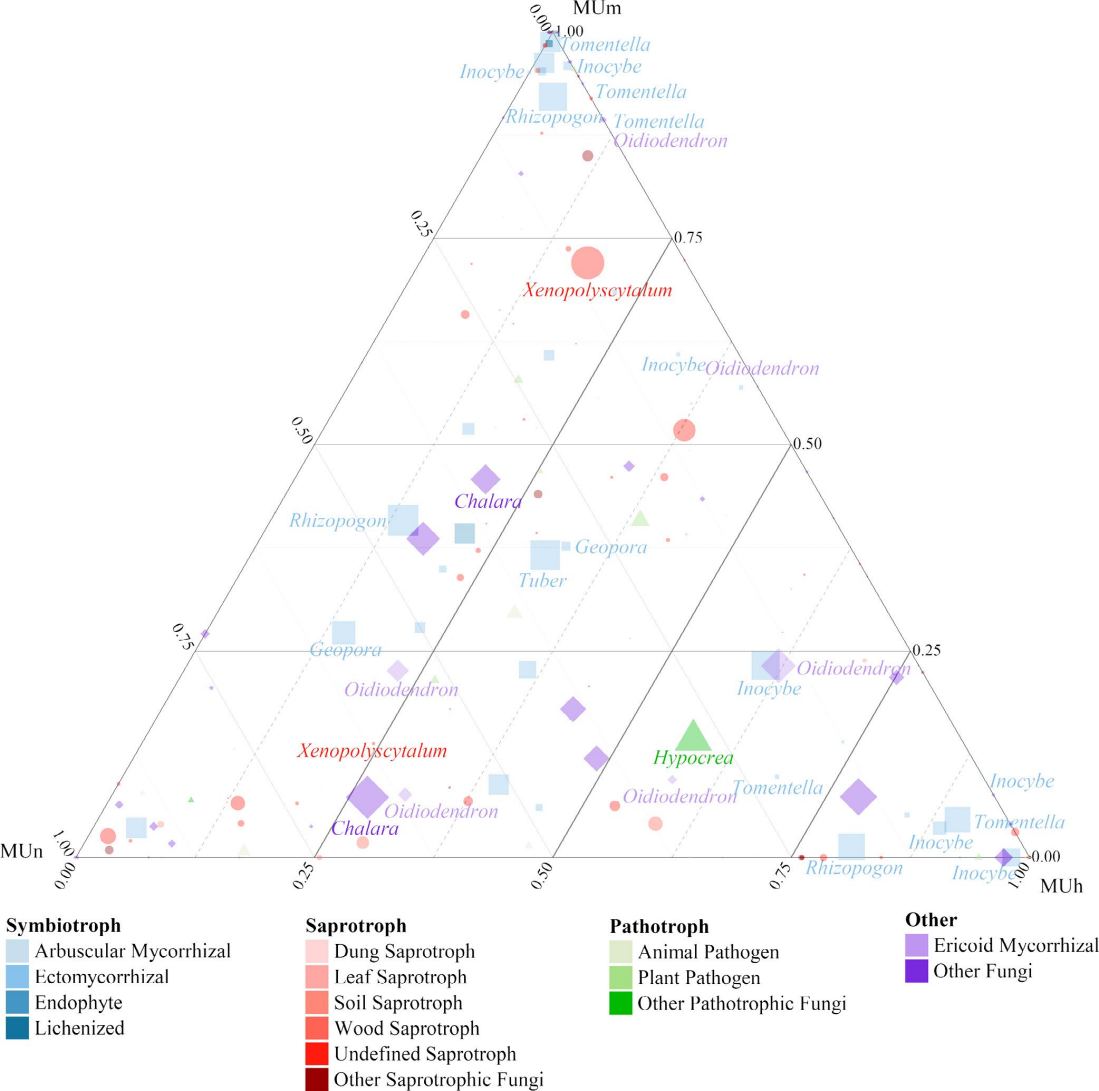
Distribution of fungal OTUs among age groups in a ternary plot. A circle represents an OTU, with the circle size corresponding to the abundance (number of sequences) of the OTU. The color indicates functional groups. Genera > 1.00% are labelled

The relative abundances of different RAF functional groups showed varying trends with increasing stand age (Table S2). The major functional group was the symbiotroph, mainly comprised of ECM fungi. In mature forests, the relative abundance of saprotrophs increased sharply, while that of pathotrophs declined, with increasing forest age. The patterns of fungal communities in three stand age were showed in a ternary plot (Figure 2), which were obtained analyzing of composition of OTUs and their abundances. The dominant genera among all plots were mostly ECM fungi (e.g., *Rhizopogon*, *Tuber*, *Inocybe*, *Tomentella*, *Geopora*, *Amphinema*, and *Hebeloma*), particularly in nearly mature and mature forests. Furthermore, it showed that several fungi with combined trophic modes (e.g., *Chalara* and *Oidiodendron*) and saprotrophs (e.g., *Xenopolyscytalum* and *Talaromyces*), and only one fungus, belonged to pathotroph (*Hypocrea*).

### 3.3 Factors affecting fungal diversity and functional group

**Figure 3:**
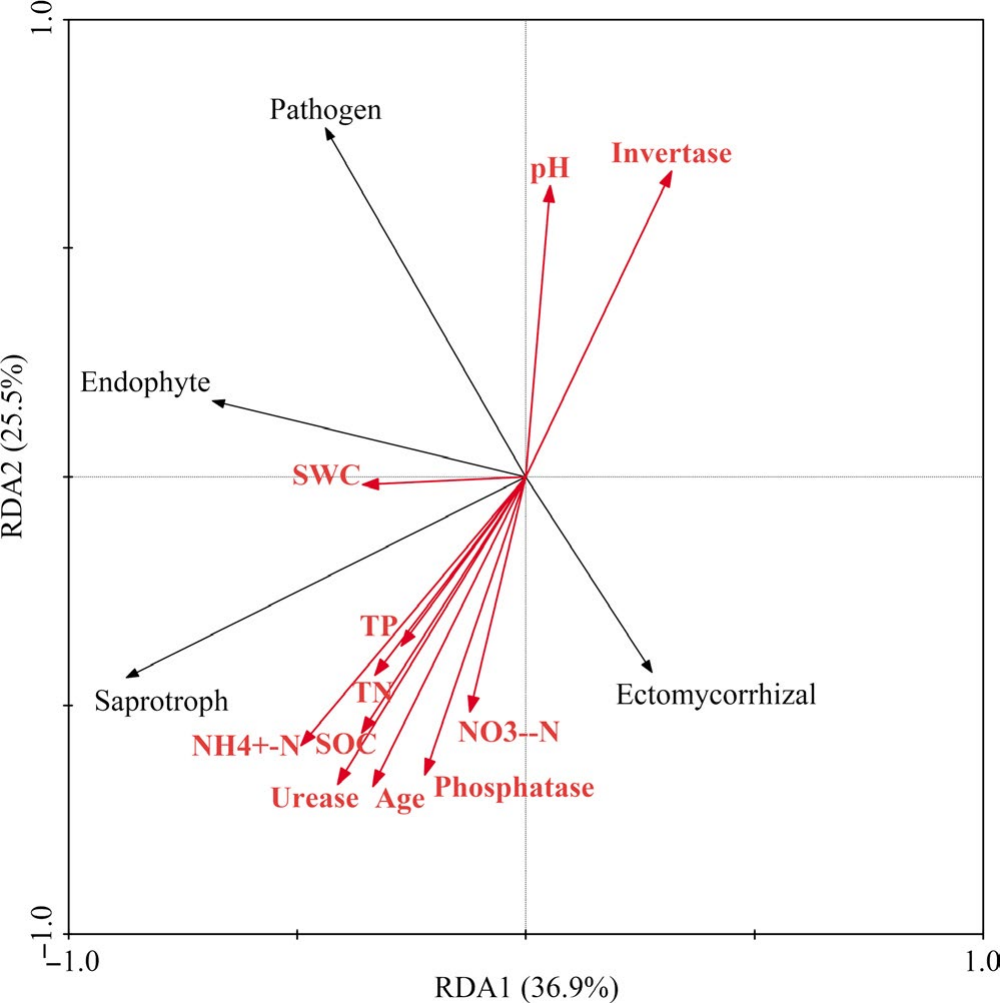
The redundancy analysis (RDA) of the RAF functional groups with soil properties and soil enzyme activities in different age groups. Arrows indicate the direction and magnitude of environmental properties associated with the trophic mode. NH_4_^+^‐N, Nitrate nitrogen; NO_3_^−^‐N, Ammonium nitrogen; SOC, soil organic carbon; SWC, soil water content; TN, total nitrogen; TP, total phosphorus

Soil properties and soil enzyme activities did not exhibit any significant correlations with fungal diversity and richness (Table 4). However, the Mantel analysis revealed that the RAF community was significantly correlated with soil enzyme activities, rather than with soil properties (Table S3). In the RDA of RAF and environmental factors, all soil indicators explained 71.4% of the variation in functional groups. Of these, urease (19.7%, *p* < .05), NH_4_^+^‐N (19.5%, *p* < .05), age (17.9%, *p* < .05), and invertase (17.2%, *p* < .05) were shown to have a greater impact on the functional group composition, compared to other variables (Figure 3). Saprotrophs were chiefly affected by SOC and invertase, while pathotrophs were mainly affected by urease and NH_4_^+^‐N.

**Table 4:** Spearman's rank correlation analysis for RAF diversity, RAF richness, soil properties, and soil enzyme activities

	Shannon		Simpson		Pielou		Richness	
	Coefficients	*p*	Coefficients	*p*	Coefficients	*p*	Coefficients	*p*
Age	0.151	.591	0.227	.416	0.094	.738	−0.265	.341
pH	−0.089	.752	−0.082	.771	−0.111	.694	0.175	.533
SWC	0.214	.443	0.089	.752	0.189	.499	0.154	.585
TN	−0.018	.950	−0.079	.781	−0.086	.761	0.079	.781
TP	0.179	.524	0.250	.369	0.136	.630	0.064	.820
SOC	0.111	.694	0.218	.435	0.154	.585	−0.254	.362
NH_4_^+^‐N	0.179	.524	0.111	.694	0.075	.791	0.214	.443
NO_3_^−^‐N	0.350	.201	0.429	.111	0.293	.289	−0.368	.177
Invertase	−0.125	.657	−0.214	.443	−0.114	.685	0.293	.289
Urease	0.257	.355	0.321	.243	0.189	.499	−0.286	.302
Phosphatase	−0.032	.909	−0.046	.869	−0.050	.860	0.043	.879

Note

∗∗*p* < .01 and ∗*p* < .05.

Abbreviations: NH_4_^+^‐N, Nitrate nitrogen; NO_3_^−^‐N, Ammonium nitrogen; SOC, soil organic carbon; SWC, soil water content; TN, total nitrogen; TP, total porosity.

## 4 DISCUSSION

### 4.1 Variations in RAF community structure, functional groups, and diversity

Fungal succession, a complex and unpredictable process, is likely to hold species richness, and diversity does not change significantly (Jumpponen, Brown, Trappe, Cázares, & Strömmer, 2012; Martinez‐Garcia, Richardson, Tylianakis, Peltzer, & Dickie, 2015). It can explain the relative stability in OTU richness in the roots of *P. sylvestris* through different age groups. RAF of *P. sylvestris* in the Mu Us Desert were diverse, and composed mainly of Ascomycota and Basidiomycota, while members of Zygomycota, Rozellomycota, Chytridiomycota, and Glomeromycota were not as numerous. This was consistent with previous observations made in most ECM‐dependent trees, such as *Quercus* and *Salix polaris* (Kernaghan & Patriquin, 2011; Toju et al., 2013). In the present study, the species and OTUs assigned to Ascomycota were more numerous than those assigned to Basidiomycota, because the faster evolutionary rate and higher species diversity of Ascomycota render it more adaptable in an arid environment than Basidiomycota (Lutzoni et al., 2004).

The proportion of RAF genera showed strong heterogeneity in the different age groups, even though the top genera were similar in fungal community composition, and this evenness decreased with increasing stand age. In each age group, there were more than 130 genera which distributed unequally, confirming the results of previous research on intraspecific compositional diversity, even at fine‐scales (Bahram, Polme, Koljalg, & Tedersoo, 2011; Mundra et al., 2015). The nonuniformity in fungal distribution derived from stand age can be explained by vegetative growth, root growth, and root exudates. In different development stage, the utilization of nutrient substance would determine the recruitment of required fungi, and microbial growth rates must be seen in light of the root growth rates (Kuzyakov & Razavi, 2019). The components of root exudates are connected with stand age, with different root exudates directly influencing the proportion and community structure of rhizosphere microorganisms (Broeckling, Broz, Bergelson, Manter, & Vivanco, 2008; Llado, Lopez‐Mondejar, & Baldrian, 2018).

The functional group structure of the RAF of *P. sylvestris* changed dynamically with the forest age, but the interaction between the different functional groups was not clear. A “down‐up” trend was observed for both symbiotrophs and saprotrophs, while the proportion of pathotrophs continued to decline with the maturing of plantations. Most mycorrhizal fungi (especially ECM fungi) cannot decompose and utilize lignin and cellulose like saprophytes (Moore et al., 2004), and ECM fungi absorb a large amount of nutrients from the soil for the host plants (Martin, Uroz, & Barker, 2017). Therefore, we believe that the sudden increase in saprotrophs is probably related to the premature maturation of *P. sylvestris*. The amount of litter and dead tissue increases gradually with maturation, providing an organic matter pool for the saprophytes. When the proportion of pathotrophs and saprotrophs is higher than symbiotrophs, it is likely to cause plant diseases (Chu, Wang, Wang, Chen, & Tang, 2016; Millberg, Boberg, & Stenlid, 2015). It might well be a potential driver to the decline of *P. sylvestris.* The half‐mature forest was more susceptible to external disturbance, which may occur a heavier recession. With the maturing of plantations and the increase of saprotrophs, ECM fungi will be driven to improve tolerance to desiccation and defense against root pathogens (Frew, Powell, Glauser, Bennett, & Johnson, 2018).

### 4.2 Predominance of ECM fungi in RAF communities

The dominant RAF of Pinaceae are the ECM fungi, providing “positive plant‐soil feedbacks” (Tedersoo, May, & Smith, 2010). In the present study, the prominent fungal groups associated with roots of *P. sylvestris* were of ECM fungi (notably *Rhizopogon*, *Tuber*, *Inocybe*, *Tomentella*, and *Geopora*), which have also been confirmed previously by ECM ground surveys using molecular identification, at a global scale (Guo et al., 2020; Hayward, Horton, & Nunez, 2015; Long, Liu, Han, Wang, & Huang, 2016; Roy‐Bolduc, Laliberte, & Hijri, 2016). Some ECM fungi have strong environmental adaptability and can establish relatively stable symbiotic relationships with hosts. At the same time, the interaction between stand age and ECM fungi was reflected in changes in tree health, owing to the reciprocity between hosts and ECM fungi. Undermining the health affects the diversity and community structure of fungi by changing the supply of ectomycorrhizal nutrients (Koide, Fernandez, & Petprakob, 2011).

*Rhizopogon*, as a predominant member of ECM fungal communities in temperate coniferous forests (Grubisha, Trappe, Molina, & Spatafora, 2002), can improve survival and drought tolerance of conifer seedlings (Van Dorp, Beiler, & Durall, 2016). *Inocybe* and *Tomentella* species are good dispersers, as they can be easily established from spores (Nara, 2009; Peay, Schubert, Nguyen, & Bruns, 2012). *Tomentella* is an ECM fungi with saprotrophic ability, and identification using FUNGuild revealed that some symbiotroph fungi also exhibited free‐living saprophytic life strategies. We speculated that these fungi may change their trophic mode with the death of plant root tissues and/or environmental changes, to maintain normal physiological activities (Baldrian, 2009). *Geopora*, widely distributed fungus that can colonize a variety of host plant species (Long et al., 2016), has been reported to be one of the dominant genera associated with *P. sylvestris* roots in arid and alkaline conditions (Ishida, Nara, Ma, Takano, & Liu, 2009), by facilitating the resistance of host plants to stress conditions.

Among other nonmycorrhizal fungi, *Chalara*, *Ilyonectria*, and *Xenopolyscytalum* were somewhat dominant, which was similar to the identification results of nonmycorrhizal RAF in *Pinus wallichiana* (Tyub et al., 2018). *Chalara* was identified as having a combined trophic mode (endophyte‐plant pathogen‐wood saprotroph) by FUNGuild. It is generally considered to be a litter saprotroph (Koukol, 2011), but certain *Chalara* species can become pathogenic, causing ash dieback (Pautasso, Aas, Queloz, & Holdenrieder, 2013). Some RAF in our study exhibited a similar phenomenon. The trophic and functional relationships between the RAF and the host plant are variable (Porras‐Alfaro & Bayman, 2011), because the instability during the endophytic developmental stages causes them to constantly change ecological strategies and behavior in different hosts with diverse living conditions (Arnold & Lutzoni, 2007). *Ilyonectria* and *Xenopolyscytalum* were indicator genera in near‐mature and mature forests (Table S4), both of which are saprotrophic fungi. Of these, *Ilyonectria* has been observed to be associated with plant disease and growth reduction in previous studies (Manici et al., 2018).

Remarkably, a few arbuscular mycorrhizal (AM) fungi were identified from the root tip samples of *P. sylvestris* in near‐mature and mature forests, as also observed in *Quercus rubra* and *Pinus densiflora*, with the preference of ECM (Dickie, Koide, & Fayish, 2001; Toju & Sato, 2018). Furthermore, *Oidiodendron*, an ericoid mycorrhizal (ERM) fungus, was also identified in all plots, and ECM‐ERM interactions have previously been reported in other nonericoid plants, such as *Quercus ilex*, *Betula papyrifera*, *Abies balsamea*, and *Picea glauca* (Bergero, Perotto, Girlanda, Vidano, & Luppi, 2000; Kernaghan & Patriquin, 2011). This spans the traditional classification model of mycorrhizal fungal colonization based on host plant species (Toju, Tanabe, & Sato, 2018), and it is more reasonable to explain the presence of AM or/and ERM fungi with fungal endophytic structures rather than the functional relationship of mycorrhizal symbiosis.

### 4.3 Effects of soil enzymes and soil properties on RAF

In general, RAF diversity is affected by climatic and/or edaphic factors, especially soil pH, moisture, and SOC (Barnes, Gast, et al., 2016b; Tedersoo et al., 2014; Toljander, Eberhardt, Toljander, Paul, & Taylor, 2006). However, the correlation between the RAF diversity of *P. sylvestris* and environmental disturbance factors was poor, which has also been shown in previous studies (Entwistle, Zak, & Edwards, 2013; Jumpponen & Jones, 2014). We speculate that the shaping of fungal diversity by environmental factors depends on the scales and categories of the ecosystem, and fungi are tolerant and resilient to climate change in some ecosystems (Zheng, Hu, Guo, Anderson, & Powell, 2017).

A previous study suggested that fungi were more reactive to soil enzyme activity than were other microbial populations (Stursova, Barta, Santruckova, & Baldrian, 2016). We observed that the RAF community was strongly associated with soil enzyme activity, particularly to enzymes involved in the carbon (invertase), nitrogen (urease), and phosphorus (phosphatases) cycles (Baldrian, 2009) (Table S5). Invertase is related to the transformation and decomposition of SOC (Sotomayor‐Ramírez, Espinoza, & Acosta‐Martínez, 2009); however, with increasing SOC in the gradually maturing *P. sylvestris* plantation, the invertase activity was significantly reduced. This opposite relationship probably resulted from the increasing saprophytic fungi, which can also decompose the SOC. Urease plays a prominent role in nitrogen mineralization, making more nitrogen available to the plants (Song et al., 2012). In our study, urease activity tends to increase with the age of *P. sylvestris*, reflecting the tendency of an increase in soil nitrogen. Pathotrophs and soil nitrogen (e.g., TN, NH_4_^+^‐N, and NO_3_^−^‐N) were found to be negatively correlated, which confirmed that effective utilization of nitrogen can improve the resistance to pathogens (Dietrich, Ploss, Heil, & Cell & Environment, 2004); however, the interplay between the two cannot be easily determined (Dordas, 2008). Phosphatase activity in rhizosphere soil is higher than in the bulk soils, because it catalyzes the release of phosphate from organic compounds (Baldrian, 2014). Previous research has shown that organic phosphorus‐targeting enzymes were of saprotrophic origin (Talbot et al., 2013; Zavišić et al., 2016), thus saprophytic fungi in the roots of *P. sylvestris* were more intimately related to phosphatase.

In contrast, many environmental factors frequently vary with changes in soil pH, which is considered to have the most definitive impact on soil fungal community composition (Barnes, Gast, McNamara, Rowe, & Bending, 2018). However, there were no significant correlations between the fungal communities in *P. sylvestris* roots and soil pH (Table S3), which may be because fungi are less sensitive to pH changes and have a wide optimum pH range, without significant inhibition of their growth in pH outside this range (Rousk et al., 2010). In addition, rhizosphere soil pH of the *P. sylvestris* plantation was around 7.4 with little pH gradient, which was not enough to affect the fungal community and function. Most soil physical and chemical properties were poorly correlated with RAF composition and trophic mode. Host roots maintain a relatively stable fend and feedback interaction with the RAF, while the rhizosphere of the individual host plant has buffering effects (Goldmann et al., 2016), so changing soil environment does not result in variations in the fungal community of roots.

Overall, we did not observe any decline in the soil fertility, but the degradation of *P. sylvestris* was evident, indicating that stand age was the chief factor. This study showed that RAF taxa and functional groups influenced the growth of *P. sylvestris* to some extent. Building the connection and circulation of the soil system, RAF and hosts are a new point to solving plantation decline. The half‐mature forest was in a vigorous period of growth, but the near‐mature forest showed signs of decline with a sharp downward trend of symbiotrophs. Furthermore, the maturing *P. sylvestris* lead to an increase in saprotrophs, and the RAF regulation mechanism required more symbiotrophs to sustain the growth of the decaying *P. sylvestris*. From a novel perspective of microbes, relevant studies are necessary to enhance our understanding of *P. sylvestris* plantation declines and provide theoretical support for *P. sylvestris* plantation management in China.

## 5 CONCLUSIONS

In the *P. sylvestris* plantation, the RAF were abundant, with various functional groups. There was heterogeneity in the distribution of fungal communities and functional guilds in the different age groups, while *Rhizopogon* dominated in each plot. Among the symbiotrophs, the ECM fungi represented a predominant RAF functional group, even if it fluctuated with the stand age. With the maturing of *P. sylvestris* plantation, the variation of RAF composition and structure caused by stand development and soil properties, especially soil enzyme activity, which can explain *P. sylvestris* degradation plantation in the Mu Us Desert. The host health status and the forest ecosystem stability can be indicated by community structure and functional groups of RAF.

## CONFLICT OF INTEREST

The authors declare no conflict of interest.

## AUTHOR CONTRIBUTIONS

Mi‐shan Guo and Guang‐lei Gao conceived and designed the experiments. Guang‐lei Gao, Mi‐shan Guo, and Yue Ren performed the experiments. Pei‐shan Zhao analyzed the data. Pei‐shan Zhao and Guang‐lei Gao drafted the manuscript. Guang‐lei Gao, Pei‐shan Zhao, and Mobeen Akhtar reviewed and edited the manuscript. Guang‐lei Gao, Ying Zhang, and Guo‐dong Ding performed supervision and finalized the manuscript.

## Data Availability

We uploaded all fungal raw sequencing data to NCBI database. The BioProject accession number was PRJNA587326 (BioSample accessions SAMN13186498, SAMN13186499 and SAMN13186500 for half‐mature forest, nearly mature forest, and mature forest, respectively).

## References

[ece36119-bib-0001] Arnold, A. E. , & Lutzoni, F. (2007). Diversity and host range of foliar fungal endophytes: Are tropical leaves biodiversity hotspots? Ecology, 88(3), 541–549. 10.1890/05-1459 17503580

[ece36119-bib-0002] Bahram, M. , Polme, S. , Koljalg, U. , & Tedersoo, L. (2011). A single European aspen (*Populus tremula*) tree individual may potentially harbour dozens of *Cenococcum geophilum* ITS genotypes and hundreds of species of ectomycorrhizal fungi. FEMS Microbiology Ecology, 75(2), 313–320. 10.1111/j.1574-6941.2010.01000.x 21114502

[ece36119-bib-0003] Baldrian, P. (2009). Ectomycorrhizal fungi and their enzymes in soils: Is there enough evidence for their role as facultative soil saprotrophs? Oecologia, 161(4), 657–660. 10.1007/s00442-009-1433-7 19685081

[ece36119-bib-0004] Baldrian, P. (2014). Distribution of extracellular enzymes in soils: Spatial heterogeneity and determining factors at various scales. Soil Science Society of America Journal, 78, 11–18. 10.2136/sssaj2013.04.0155dgs

[ece36119-bib-0005] Barnes, C. J. , Maldonado, C. , Froslev, T. G. , Antonelli, A. , & Ronsted, N. (2016a). Unexpectedly high beta‐diversity of root‐associated fungal communities in the Bolivian Andes. Frontiers in Microbiology, 7, 1377 10.3389/fmicb.2016.01377 27630629 PMC5006319

[ece36119-bib-0006] Barnes, C. J. , van der Gast, C. J. , Burns, C. A. , McNamara, N. P. , & Bending, G. D. (2016b). Temporally variable geographical distance effects contribute to the assembly of root‐associated fungal communities. Frontiers in Microbiology, 7, 195 10.3389/fmicb.2016.00195 26941720 PMC4766365

[ece36119-bib-0007] Barnes, C. J. , van der Gast, C. J. , McNamara, N. P. , Rowe, R. , & Bending, G. D. (2018). Extreme rainfall affects assembly of the root‐associated fungal community. New Phytologist, 220(4), 1172–1184. 10.1111/nph.14990 29350759 PMC6282977

[ece36119-bib-0008] Bergero, R. , Perotto, S. , Girlanda, M. , Vidano, G. , & Luppi, A. M. (2000). Ericoid mycorrhizal fungi are common root associates of a Mediterranean ectomycorrhizal plant (*Quercus ilex*). Molecular Ecology, 9(10), 1639–1649. 10.1046/j.1365-294x.2000.01059.x 11050558

[ece36119-bib-0009] Broeckling, C. D. , Broz, A. K. , Bergelson, J. , Manter, D. K. , & Vivanco, J. M. (2008). Root exudates regulate soil fungal community composition and diversity. Applied and Environmental Microbiology, 74(3), 738–744. 10.1128/AEM.02188-07 18083870 PMC2227741

[ece36119-bib-0010] Chu, H. , Wang, C. , Wang, H. , Chen, H. , & Tang, M. (2016). Pine wilt disease alters soil properties and root‐associated fungal communities in *Pinus tabulaeformis* forest. Plant and Soil, 404(1–2), 237–249. 10.1007/s11104-016-2845-x

[ece36119-bib-0011] David, A. S. , Seabloom, E. W. , & May, G. (2016). Plant host species and geographic distance affect the structure of aboveground fungal symbiont communities, and environmental filtering affects belowground communities in a coastal dune ecosystem. Microbial Ecology, 71(4), 912–926. 10.1007/s00248-015-0712-6 26626912

[ece36119-bib-0012] Dickie, I. A. , Koide, R. T. , & Fayish, A. C. J. N. P. (2001). Vesicular‐arbuscular mycorrhizal infection of *Quercus rubra* seedlings. New Phytologist, 151, 257–264. 10.1046/j.1469-8137.2001.00148.x 33873380

[ece36119-bib-0013] Dietrich, R. , Ploss, K. , Heil, M. J. P. (2004). Constitutive and induced resistance to pathogens in *Arabidopsis thaliana* depends on nitrogen supply. Plant, Cell and Environment, 27(7), 896–906. 10.1111/j.1365-3040.2004.01195.x

[ece36119-bib-0014] Dordas, C. (2008). Role of nutrients in controlling plant diseases in sustainable agriculture. A Review. Agronomy for Sustainable Development, 28(1), 33–46. 10.1051/agro:2007051

[ece36119-bib-0015] Entwistle, E. M. , Zak, D. R. , & Edwards, I. P. (2013). Long‐term experimental nitrogen deposition alters the composition of the active fungal community in the forest floor. Soil Science Society of America Journal, 77(5), 1648–1658. 10.2136/sssaj2013.05.0179

[ece36119-bib-0016] Fisher, M. C. , Henk, D. A. , Briggs, C. J. , Brownstein, J. S. , Madoff, L. C. , McCraw, S. L. , & Gurr, S. J. (2012). Emerging fungal threats to animal, plant and ecosystem health. Nature, 484(7393), 186–194. 10.1038/nature10947 22498624 PMC3821985

[ece36119-bib-0017] Frew, A. , Powell, J. R. , Glauser, G. , Bennett, A. E. , & Johnson, S. N. (2018). Mycorrhizal fungi enhance nutrient uptake but disarm defences in plant roots, promoting plant‐parasitic nematode populations. Soil Biology and Biochemistry, 126, 123–132. 10.1016/j.soilbio.2018.08.019

[ece36119-bib-0018] Gao, G. L. , Ding, G. D. , Zhou, Y. Y. , Wu, B. , Zhang, Y. Q. , Qin, S. G. , … Liu, Y. D. (2014). Fractal approach to estimating changes in soil properties following the establishment of *Caragana korshinskii* shelterbelts in Ningxia, NW China. Ecological Indicators, 43, 236–243. 10.1016/j.ecolind.2014.03.00

[ece36119-bib-0019] Goldmann, K. , Schröter, K. , Pena, R. , Schöning, I. , Schrumpf, M. , Buscot, F. , … Wubet, T. (2016). Divergent habitat filtering of root and soil fungal communities in temperate beech forests. Scientific Reports, 6, 31439 10.1038/srep31439 27511465 PMC4980589

[ece36119-bib-0020] Gong, D. Y. , Shi, P. J. , & Wang, J. A. (2004). Daily precipitation changes in the semi‐arid region over northern China. Journal of Arid Environments, 59(4), 771–784. 10.1016/j.jaridenv.2004.02.006

[ece36119-bib-0021] Grubisha, L. C. , Trappe, J. M. , Molina, R. , & Spatafora, J. W. (2002). Biology of the ectomycorrhizal genus *Rhizopogon*. VI. Re‐examination of infrageneric relationships inferred from phylogenetic analyses of ITS sequences. Mycologia, 94(4), 607–619. 10.1080/15572536.2003.11833189 21156534

[ece36119-bib-0022] Guo, M. S. , Ding, G. D. , Gao, G. L. , Zhang, Y. , Cao, H. Y. , & Ren, Y. (2020). Community composition of ectomycorrhizal fungi associated with *Pinus sylvestris* var. *mongolica* plantations of various ages in the Horqin Sandy Land. Ecological Indicators, 110, 105860 10.1016/j.ecolind.2019.105860

[ece36119-bib-0023] Hayward, J. , Horton, T. R. , & Nunez, M. A. (2015). Ectomycorrhizal fungal communities coinvading with Pinaceae host plants in Argentina: Gringos bajo el bosque. New Phytologist, 208(2), 497–506. 10.1111/nph.13453 25963605

[ece36119-bib-0024] Horn, S. , Hempel, S. , Verbruggen, E. , Rillig, M. C. , & Caruso, T. (2017). Linking the community structure of arbuscular mycorrhizal fungi and plants: A story of interdependence? ISME Journal, 11(6), 1400–1411. 10.1038/ismej.2017.5 28244977 PMC5437357

[ece36119-bib-0025] Ishida, T. A. , Nara, K. , Ma, S. , Takano, T. , & Liu, S. (2009). Ectomycorrhizal fungal community in alkaline‐saline soil in northeastern China. Mycorrhiza, 19(5), 329–335. 10.1007/s00572-008-0219-9 19104846

[ece36119-bib-0026] Jumpponen, A. , Brown, S. P. , Trappe, J. M. , Cázares, E. , & Strömmer, R. (2012). Twenty years of research on fungal‐plant interactions on Lyman Glacier forefront‐lessons learned and questions yet unanswered. Fungal Ecology, 5(4), 430–442. 10.1016/j.funeco.2012.01.002

[ece36119-bib-0027] Jumpponen, A. , & Jones, K. L. (2014). Tallgrass prairie soil fungal communities are resilient to climate change. Fungal Ecology, 10, 44–57. 10.1016/j.funeco.2013.11.003

[ece36119-bib-0028] Kernaghan, G. , & Patriquin, G. (2011). Host associations between fungal root endophytes and boreal trees. Microbial Ecology, 62(2), 460–473. 10.1007/s00248-011-9851-6 21475991

[ece36119-bib-0029] Koide, R. T. , Fernandez, C. , & Petprakob, K. (2011). General principles in the community ecology of ectomycorrhizal fungi. Annals of Forest Science, 68(1), 45–55. 10.1007/s13595-010-0006-6

[ece36119-bib-0030] Kolaříková, Z. , Kohout, P. , Krüger, C. , Janoušková, M. , Mrnka, L. , & Rydlová, J. (2017). Root‐associated fungal communities along a primary succession on a mine spoil: Distinct ecological guilds assemble differently. Soil Biology and Biochemistry, 113, 143–152. 10.1016/j.soilbio.2017.06.004

[ece36119-bib-0031] Koukol, O. (2011). New species of *Chalara* occupying coniferous needles. Fungal Diversity, 49(1), 75–91. 10.1007/s13225-011-0092-2

[ece36119-bib-0032] Kuzyakov, Y. , & Razavi, B. S. (2019). Rhizosphere size and shape: Temporal dynamics and spatial stationarity. Soil Biology and Biochemistry, 135, 343–360. 10.1016/j.soilbio.2019.05.011

[ece36119-bib-0033] Kyaschenko, J. , Clemmensen, K. E. , Hagenbo, A. , Karltun, E. , & Lindahl, B. D. (2017). Shift in fungal communities and associated enzyme activities along an age gradient of managed *Pinus sylvestris* stands. ISME Journal, 11(4), 863–874. 10.1038/ismej.2016.184 28085155 PMC5364365

[ece36119-bib-0034] Li, S. , Shakoor, A. , Wubet, T. , Zhang, N. , Liang, Y. , & Ma, K. (2018). Fine‐scale variations of fungal community in a heterogeneous grassland in Inner Mongolia: Effects of the plant community and edaphic parameters. Soil Biology and Biochemistry, 122, 104–110. 10.1016/j.soilbio.2018.04.007

[ece36119-bib-0035] Liu, Y. , Bao, G. , Song, H. , Cai, Q. , & Sun, J. (2009). Precipitation reconstruction from Hailar pine (*Pinus sylvestris* var. *mongolica*) tree rings in the Hailar region, Inner Mongolia, China back to 1865 AD. Palaeogeography, Palaeoclimatology, Palaeoecology, 282, (1–4), 81–87 10.1016/j.palaeo.2009.08.012

[ece36119-bib-0036] Liu, Y. , Mao, L. , Li, J. , Shi, G. , Jiang, S. , Ma, X. , … Feng, H. (2014). Resource availability differentially drives community assemblages of plants and their root‐associated arbuscular mycorrhizal fungi. Plant and Soil, 386(1–2), 341–355. 10.1007/s11104-014-2261-z

[ece36119-bib-0037] Llado, S. , Lopez‐Mondejar, R. , & Baldrian, P. (2018). Drivers of microbial community structure in forest soils. Applied Microbiology and Biotechnology, 102(10), 4331–4338. 10.1007/s00253-018-8950-4 29600493

[ece36119-bib-0038] Long, D. , Liu, J. , Han, Q. , Wang, X. , & Huang, J. (2016). Ectomycorrhizal fungal communities associated with *Populus simonii* and *Pinus tabuliformis* in the hilly‐gully region of the Loess Plateau, China. Scientific Reports, 6, 24336 10.1038/srep24336 27063338 PMC4827030

[ece36119-bib-0039] Lutzoni, F. , Kauff, F. , Cox, C. J. , McLaughlin, D. , Celio, G. , Dentinger, B. , … Vilgalys, R. (2004). Assembling the fungal tree of life: Progress, classification, and evolution of subcellular traits. American Journal of Botany, 91(10), 1446–1480. 10.3732/ajb.91.10.1446 21652303

[ece36119-bib-0040] Manici, L. M. , Kelderer, M. , Caputo, F. , Saccà, M. L. , Nicoletti, F. , Topp, A. R. , & Mazzola, M. (2018). Involvement of *Dactylonectria* and *Ilyonectria* spp. in tree decline affecting multi‐generation apple orchards. Plant and Soil, 425(1‐2), 217–230. 10.1007/s11104-018-3571-3

[ece36119-bib-0041] Martin, F. M. , Uroz, S. , & Barker, D. G. (2017). Ancestral alliances: Plant mutualistic symbioses with fungi and bacteria. Science, 356(6340), eaad4501 10.1126/science.aad4501 28546156

[ece36119-bib-0042] Martinez‐Garcia, L. B. , Richardson, S. J. , Tylianakis, J. M. , Peltzer, D. A. , & Dickie, I. A. (2015). Host identity is a dominant driver of mycorrhizal fungal community composition during ecosystem development. New Phytologist, 205(4), 1565–1576. 10.1111/nph.13226 25640965

[ece36119-bib-0043] Millberg, H. , Boberg, J. , & Stenlid, J. (2015). Changes in fungal community of Scots pine (*Pinus sylvestris*) needles along a latitudinal gradient in Sweden. Fungal Ecology, 17, 126–139. 10.1016/j.funeco.2015.05.012

[ece36119-bib-0044] Moore, J. C. , Berlow, E. L. , Coleman, D. C. , Ruiter, P. C. , Dong, Q. , Hastings, A. , … Wall, D. H. (2004). Detritus, trophic dynamics and biodiversity. Ecology Letters, 7(7), 584–600. 10.1111/j.1461-0248.2004.00606.x

[ece36119-bib-0045] Mundra, S. , Bahram, M. , Tedersoo, L. , Kauserud, H. , Halvorsen, R. , & Eidesen, P. B. (2015). Temporal variation of *Bistorta vivipara*‐associated ectomycorrhizal fungal communities in the High Arctic. Molecular Ecology, 24(24), 6289–6302. 10.1111/mec.13458 26547806

[ece36119-bib-0046] Nara, K. (2009). Spores of ectomycorrhizal fungi: Ecological strategies for germination and dormancy. New Phytologist, 181(2), 245–248. 10.1111/j.1469-8137.2008.02691.x 19121026

[ece36119-bib-0047] Nguyen, N. H. , Song, Z. , Bates, S. T. , Branco, S. , Tedersoo, L. , Menke, J. , … Kennedy, P. G. (2016). FUNGuild: An open annotation tool for parsing fungal community datasets by ecological guild. Fungal Ecology, 20, 241–248. 10.1016/j.funeco.2015.06.006

[ece36119-bib-0048] Pautasso, M. , Aas, G. , Queloz, V. , & Holdenrieder, O. (2013). European ash (*Fraxinus excelsior*) dieback – A conservation biology challenge. Biological Conservation, 158, 37–49. 10.1016/j.biocon.2012.08.026

[ece36119-bib-0049] Peay, K. G. , Baraloto, C. , & Fine, P. V. (2013). Strong coupling of plant and fungal community structure across western Amazonian rainforests. ISME Journal, 7(9), 1852–1861. 10.1038/ismej.2013.66 23598789 PMC3749505

[ece36119-bib-0050] Peay, K. G. , Schubert, M. G. , Nguyen, N. H. , & Bruns, T. D. (2012). Measuring ectomycorrhizal fungal dispersal: Macroecological patterns driven by microscopic propagules. Molecular Ecology, 21(16), 4122–4136. 10.1111/j.1365-294X.2012.05666.x 22703050

[ece36119-bib-0051] Porras‐Alfaro, A. , & Bayman, P. (2011). Hidden fungi, emergent properties: Endophytes and microbiomes. Annual Review of Phytopathology, 49, 291–315. 10.1146/annurev-phyto-080508-081831 19400639

[ece36119-bib-0052] Rousk, J. , Bååth, E. , Brookes, P. C. , Lauber, C. L. , Lozupone, C. , Caporaso, J. G. , … Fierer, N. (2010). Soil bacterial and fungal communities across a pH gradient in an arable soil. ISME Journal, 4(10), 1340–1351. 10.1038/ismej.2010.58 20445636

[ece36119-bib-0053] Roy‐Bolduc, A. , Laliberte, E. , & Hijri, M. (2016). High richness of ectomycorrhizal fungi and low host specificity in a coastal sand dune ecosystem revealed by network analysis. Ecology and Evolution, 6(1), 349–362. 10.1002/ece3.1881 26811798 PMC4716518

[ece36119-bib-0054] Song, L. , Zhu, J. , Li, M. , & Zhang, J. (2016). Water use patterns of *Pinus sylvestris* var. *mongolica* trees of different ages in a semiarid sandy lands of Northeast China. Environmental and Experimental Botany, 129, 94–107. 10.1016/j.envexpbot.2016.02.006

[ece36119-bib-0055] Song, L. , Zhu, J. , Li, M. , Zhang, J. , Zheng, X. , & Wang, K. (2018). Canopy transpiration of *Pinus sylvestris* var. *mongolica* in a sparse wood grassland in the semiarid sandy region of Northeast China. Agricultural and Forest Meteorology, 250–251, 192–201. 10.1016/j.agrformet.2017.12.260

[ece36119-bib-0056] Song, L. , Zhu, J. , Yan, Q. , Li, M. , & Yu, G. (2015). Comparison of intrinsic water use efficiency between different aged *Pinus sylvestris* var. *mongolica* wide windbreaks in semiarid sandy land of northern China. Agroforestry Systems, 89(3), 477–489. 10.1007/s10457-014-9784-4

[ece36119-bib-0057] Song, Y. , Song, C. , Yang, G. , Miao, Y. , Wang, J. , & Guo, Y. (2012). Changes in labile organic carbon fractions and soil enzyme activities after marshland reclamation and restoration in the Sanjiang Plain in northeast China. Environmental Management, 50(3), 418–426. 10.1007/s00267-012-9890-x 22744158

[ece36119-bib-0058] Sotomayor‐Ramírez, D. , Espinoza, Y. , & Acosta‐Martínez, V. (2009). Land use effects on microbial biomass C, β‐glucosidase and β‐glucosaminidase activities, and availability, storage, and age of organic C in soil. Biology and Fertility of Soils, 45(5), 487–497. 10.1007/s00374-009-0359-x

[ece36119-bib-0059] Spake, R. , van der Linde, S. , Newton, A. C. , Suz, L. M. , Bidartondo, M. I. , & Doncaster, C. P. (2016). Similar biodiversity of ectomycorrhizal fungi in set‐aside plantations and ancient old‐growth broadleaved forests. Biological Conservation, 194, 71–79. 10.1016/j.biocon.2015.12.003 26917858 PMC4730558

[ece36119-bib-0060] Stursova, M. , Barta, J. , Santruckova, H. , & Baldrian, P. (2016). Small‐scale spatial heterogeneity of ecosystem properties, microbial community composition and microbial activities in a temperate mountain forest soil. FEMS Microbiology Ecology, 92(12), fiw185 10.1093/femsec/fiw185 27604254

[ece36119-bib-0061] Talbot, J. M. , Bruns, T. D. , Smith, D. P. , Branco, S. , Glassman, S. I. , Erlandson, S. , … Peay, K. G. (2013). Independent roles of ectomycorrhizal and saprotrophic communities in soil organic matter decomposition. Soil Biology and Biochemistry, 57, 282–291. 10.1016/j.soilbio.2012.10.004

[ece36119-bib-0062] Tedersoo, L. , Bahram, M. , Põlme, S. , Kõljalg, U. , Yorou, N. S. , Wijesundera, R. , … Abarenkov, K. (2014). Global diversity and geography of soil fungi. Science, 346(6213), 1256688 10.1126/science.1256688 25430773

[ece36119-bib-0063] Tedersoo, L. , May, T. W. , & Smith, M. E. (2010). Ectomycorrhizal lifestyle in fungi: Global diversity, distribution, and evolution of phylogenetic lineages. Mycorrhiza, 20(4), 217–263. 10.1007/s00572-009-0274-x 20191371

[ece36119-bib-0064] Toju, H. , & Sato, H. (2018). Root‐Associated fungi shared between arbuscular mycorrhizal and ectomycorrhizal conifers in a temperate forest. Frontiers in Microbiology, 9, 433 10.3389/fmicb.2018.00433 29593682 PMC5858530

[ece36119-bib-0065] Toju, H. , Tanabe, A. S. , & Sato, H. (2018). Network hubs in root‐associated fungal metacommunities. Microbiome, 6(1), 116 10.1186/s40168-018-0497-1 29935536 PMC6015470

[ece36119-bib-0066] Toju, H. , Yamamoto, S. , Sato, H. , Tanabe, A. S. , Gilbert, G. S. , & Kadowaki, K. (2013). Community composition of root‐associated fungi in a *Quercus*‐dominated temperate forest: “codominance” of mycorrhizal and root‐endophytic fungi. Ecology and Evolution, 3(5), 1281–1293. 10.1002/ece3.546 23762515 PMC3678483

[ece36119-bib-0067] Toljander, J. F. , Eberhardt, U. , Toljander, Y. K. , Paul, L. R. , & Taylor, A. F. (2006). Species composition of an ectomycorrhizal fungal community along a local nutrient gradient in a boreal forest. New Phytologist, 170(4), 873–883. 10.1111/j.1469-8137.2006.01718.x 16684245

[ece36119-bib-0068] Tyub, S. , Kamili, A. N. , Reshi, Z. A. , Rashid, I. , Mokhdomi, T. A. , Bukhari, S. , … Qadri, R. A. (2018). Root‐associated fungi of *Pinus wallichiana* in Kashmir Himalaya. Canadian Journal of Forest Research, 48(8), 923–929. 10.1139/cjfr-2018-0084

[ece36119-bib-0069] Van Dorp, C. H. , Beiler, K. J. , & Durall, D. M. (2016). Dominance of a Rhizopogon sister species corresponds to forest age structure. Mycorrhiza, 26(2), 169–175. 10.1007/s00572-015-0660-5 26265310

[ece36119-bib-0070] Wehner, J. , Powell, J. R. , Muller, L. A. H. , Caruso, T. , Veresoglou, S. D. , Hempel, S. , … van der Heijden, M. (2014). Determinants of root‐associated fungal communities within Asteraceae in a semi‐arid grassland. Journal of Ecology, 102(2), 425–436. 10.1111/1365-2745.12197

[ece36119-bib-0071] Wei, K. , & Wang, L. (2013). Reexamination of the aridity conditions in arid northwestern China for the last decade. Journal of Climate, 26(23), 9594–9602. 10.1175/jcli-d-12-00605.1

[ece36119-bib-0072] Zavišić, A. , Nassal, P. , Yang, N. , Heuck, C. , Spohn, M. , Marhan, S. , … Polle, A. (2016). Phosphorus availabilities in beech (*Fagus sylvatica* L.) forests impose habitat filtering on ectomycorrhizal communities and impact tree nutrition. Soil Biology and Biochemistry, 98, 127–137. 10.1016/j.soilbio.2016.04.006

[ece36119-bib-0073] Zheng, X. , Zhu, J. J. , Yan, Q. L. , & Song, L. N. (2012). Effects of land use changes on the groundwater table and the decline of *Pinus sylvestiris* var. *mongolica* plantations in southern Horqin Sandy Land, Northeast China. Agricultural Water Management, 109, 94–106. 10.1016/j.agwat.2012.02.010

[ece36119-bib-0074] Zheng, Y. , Hu, H. W. , Guo, L. D. , Anderson, I. C. , & Powell, J. R. (2017). Dryland forest management alters fungal community composition and decouples assembly of root‐ and soil‐associated fungal communities. Soil Biology and Biochemistry, 109, 14–22. 10.1016/j.soilbio.2017.01.024

[ece36119-bib-0075] Zhu, J. J. , Fan, Z. P. , Zeng, D. H. , Jiang, F. Q. , & Matsuzaki, T. (2003). Comparison of stand structure and growth between artificial and natural forests of *Pinus sylvestiris* var. *mongolica* on sandy land. Journal of Forestry Research, 14(2), 103–111. 10.1007/bf02856774

