# Supplementary material for: Community structure and functional group of root‐associated Fungi of Pinus sylvestris var. mongolica across stand ages in the Mu Us Desert
Source: Ecol Evol. 2020 Feb 19;10(6):3032–42. doi: 10.1002/ece3.6119 (PMC7083681; doi:10.1002/ece3.6119)
Supplement: Supplementary file 1 [file ECE3-10-3032-s001.docx]

Appendix S1

| **Table S1** Soil properties and soil enzyme activities of different age groups | | | |
| --- | --- | --- | --- |
|  | **MUh** | **MUn** | **MUm** |
| pH | 7.58±0.19 a | 7.51±0.04 a | 7.27±0.15 b |
| SWC (%) | 3.66±0.56 a | 2.88±0.17 b | 3.46±0.46 a |
| TN (g/kg) | 0.12±0.02 b | 0.09±0.01 c | 0.17±0.02 a |
| TP (g/kg) | 0.30±0.02 b | 0.37±0.01 a | 0.37±0.04 a |
| SOC (g/kg) | 1.16±0.21 c | 2.38±0.30 b | 3.23±0.85 a |
| NH_4_^+^ -N (mg/kg) | 1.03±0.06 b | 0.94±0.10 b | 1.56±0.10 a |
| NO_3_^-^ -N (mg/kg) | 2.12±0.02 c | 4.06±0.08 b | 4.30±0.04 a |
| Invertase (U/g) | 66.88±0.16 a | 48.26±1.38 b | 17.40±0.24 c |
| Urease (U/g) | 289.97±16.48 c | 319.03±8.44 b | 555.66±16.01 a |
| Phosphatase (U/g) | 0.59±0.03 b | 0.60±0.01 b | 0.69±0.03 a |
| SWC: soil water content; TN: total nitrogen; TP: total phosphorus; SOC: soil organic carbon; NH_4_^+^-N: Nitrate nitrogen; NO_3_^-^-N: Ammonium nitrogen. Values are mean ± standard error. Same minuscule alphabet in the row indicate non-significant divergence. | | | |

| **Table** **S2.** Functional groups and relative abundance of RAF in different age groups. | | | | |
| --- | --- | --- | --- | --- |
| **T****rophic mode** | **Guild** | **MUh (%)** | **MUn (%)** | **MUm (%)** |
| Symbiotroph | Arbuscular mycorrhizal | - | 0.0038 | 0.0039 |
|  | Ectomycorrhizal | 52.9456 | 37.4462 | 60.0772 |
|  | Endophyte | 0.5321 | 0.9433 | 0.9681 |
|  | Lichenized | 0.0022 | 0.0057 | 0.3421 |
|  | Total | 53.4799 | 38.3989 | 61.3912 |
| Saprotroph | Dung saprotroph | 0.0280 | - | - |
|  | Leaf saprotroph | - | - | 0.0235 |
|  | Soil saprotroph | 0.1698 | 0.1026 | 0.0999 |
|  | Wood saprotroph | 0.4074 | 0.5175 | 0.1630 |
|  | Undefined saprotroph | 7.0124 | 5.2096 | 23.2008 |
|  | Other saprotrophic fungi | 0.3165 | 0.2940 | 0.6080 |
|  | Total | 7.9341 | 6.1237 | 24.0952 |
| Pathotroph | Animal pathogen | 0.3358 | 0.6345 | 0.1594 |
|  | Plant pathogen | 0.6521 | 0.3774 | 0.9268 |
|  | Other pathotrophic fungi | 13.0248 | 6.1951 | 3.2141 |
|  | Total | 14.0127 | 7.2070 | 4.3003 |
| Other | Ericoid mycorrhizal | 3.5155 | 0.9261 | 1.4382 |
|  | Other fungi | 21.0578 | 27.5443 | 8.7751 |
|  | Total | 24.5733 | 28.4704 | 10.2133 |

| **Table S3.** Mantel test analysis of the fungal community and soil properties and soil enzyme activities in different age groups. | | |
| --- | --- | --- |
|  | ***r*** | ***P*** |
| Age | 0.239 | **0.017** |
| pH | 0.125 | 0.173 |
| SWC | 0.182 | 0.079 |
| TN | 0.067 | 0.275 |
| TP | 0.101 | 0.210 |
| SOM | 0.208 | 0.064 |
| NH_4_^+^-N | 0.216 | 0.044 |
| NO_3_^-^-N | 0.104 | 0.173 |
| Invertase | 0.231 | **0.017** |
| Urease | 0.229 | **0.034** |
| Phosphatase | 0.285 | **0.009** |
| SWC: soil water content, TN: total nitrogen, TP: total phosphorus, SOC: soil organic carbon, NH_4_^+^-N: Nitrate nitrogen，NO_3_^-^-N: Ammonium nitrogen. Bold values indicate significant divergence. The correlation (*r*) and significance (*P*) were determined by Mantel tests based on 999 permutations between community structure (Bray-Curtis dissimilarity) and environmental variables (Euclidean distance). | | |

| **Table S4.** Indicator genera of RAF in different age groups. | | | | | | |
| --- | --- | --- | --- | --- | --- | --- |
|  | **Reflect age group** | **Indicator value** | ***P*** | **Trophic mode** | **Guild** | **Confidence ranking** |
| *Ilyonectria* | MUn | 0.787 | 0.036 | Saprotroph | Undefined Saprotroph | Possible |
| *Xenopolyscytalum* | MUm | 0.827 | 0.007 | Saprotroph | Undefined Saprotroph | Probable |
| Only genera >1.0% were analyzed. | | | | | | |

| **Table S5.** Spearman’s rank correlation analysis for soil properties and soil enzyme activities. | | | | | | |
| --- | --- | --- | --- | --- | --- | --- |
|  | **Invertase** | | **Urease** | | **Phosphatase** | |
|  | Coefficients | *P* | Coefficients | *P* | Coefficients | *P* |
| Age | -0.945^**^ | 0.000 | 0.926^**^ | 0.000 | 0.756^**^ | 0.001 |
| PH | 0.618^*^ | 0.014 | -0.718^**^ | 0.003 | -0.636^*^ | 0.011 |
| SWC | -0.089 | 0.752 | 0.079 | 0.781 | -0.032 | 0.909 |
| TN | -0.450 | 0.092 | 0.457 | 0.087 | 0.582^*^ | 0.023 |
| TP | -0.650^**^ | 0.009 | 0.589^*^ | 0.021 | 0.379 | 0.164 |
| SOC | -0.857^**^ | 0.000 | 0.832^**^ | 0.000 | 0.571^*^ | 0.026 |
| NH_4_^+^-N | -0.561^*^ | 0.030 | 0.650^**^ | 0.009 | 0.525^*^ | 0.044 |
| NO_3_^-^-N | -0.893^**^ | 0.000 | 0.907^**^ | 0.000 | 0.625^*^ | 0.013 |
| SWC: soil water content; TN: total nitrogen; TP: total phosphorus; SOC: soil organic carbon; NH_4_^+^-N: Nitrate nitrogen; NO_3_^-^-N: Ammonium nitrogen; and, ∗∗*P* < 0.01, ∗*P* < 0.05. | | | | | | |
